# Laser Spot Tracking Based on Modified Circular Hough Transform and Motion Pattern Analysis

**DOI:** 10.3390/s141120112

**Published:** 2014-10-27

**Authors:** Damir Krstinić, Ana Kuzmanić Skelin, Ivan Milatić

**Affiliations:** Faculty of Electrical Engineering, Mechanical Engineering and Naval Architecture, University of Split, R. Boškovića 32, Split 21000, Croatia; E-Mails: damir.krstinic@fesb.hr (D.K.); imilatic@fesb.hr (I.M.)

**Keywords:** laser pointer detection, Hough transform, tracking

## Abstract

Laser pointers are one of the most widely used interactive and pointing devices in different human-computer interaction systems. Existing approaches to vision-based laser spot tracking are designed for controlled indoor environments with the main assumption that the laser spot is very bright, if not the brightest, spot in images. In this work, we are interested in developing a method for an outdoor, open-space environment, which could be implemented on embedded devices with limited computational resources. Under these circumstances, none of the assumptions of existing methods for laser spot tracking can be applied, yet a novel and fast method with robust performance is required. Throughout the paper, we will propose and evaluate an efficient method based on modified circular Hough transform and Lucas–Kanade motion analysis. Encouraging results on a representative dataset demonstrate the potential of our method in an uncontrolled outdoor environment, while achieving maximal accuracy indoors. Our dataset and ground truth data are made publicly available for further development.

## 1. Introduction

A laser spot is used as input information in a number of laser pointer—camera sensing systems, such as interactive interfaces [1–4], laser guided robots [5–7], assistive technology application [8] and range measurements [9]. Different methods have been proposed for detection and tracking of a laser spot. In a domotic control system [8], template matching combined with fuzzy rules is proposed for the detection of a laser spot. The reported success rate is 69% with tuned fuzzy rule parameters on the test set of 105 images (the size of the overall image set is 210). In [10], a laser spot tracking algorithm based on a subdivision mesh is proposed. Although sub-pixel accuracy is achieved, it is evaluated in an environment with controlled light. In [1], a laser spot detection-based computer interface is presented, where the method is based on perceptron learning. Insufficient testing data, as declared by the authors, leaves the applicability of the method for real-world applications inconclusive. In [11], fractional Fourier transform is used for laser detection and tracking. Favorable results are given in comparison to Kalman filtering tracking and mean-shift tracking. However, results are obtained on a small test set of close-up images of a laser spot. Among these most prominent examples of approaches, only the work of Chavez *et al.* [8] systematically evaluates the performance of the detection method, while in [1,10,11], the results are usually tested on a small dataset. Furthermore, a substantial amount of literature proposes methods for laser spot detection and tracking as a part of larger systems. Here, laser spots detected during movement are used to create gestures without evaluating or reporting the detection or tracking method. For example, in [5] and [6], a laser pointer is used as a control interface for guiding robots. In [5], a method based on green color segmentation and brightest spot detection is applied, while in [6], a method based on the optical flow pattern of the laser spot is proposed to extract different shapes from laser traces, which are associated with a set of commands for robot steering. It should be noted that in [5,6], tracking is achieved as a temporal collection of detections.

The rest of the surveyed methods are described in the relatively large body of literature devoted to the development of interfaces based on projection screens [12–15]. In this application domain, the authors deploy methods that are generally based on template matching, color segmentation and brightness thresholding, all of which have their significant disadvantages when operated under uncontrolled conditions. Template matching is known to be inappropriate for the problem of small target tracking, where the target occupies only a few pixels in an image, while color segmentation and brightness thresholding have an underlying assumption that the laser spot has the specific color and/or occurs as the brightest spot in images, which may not hold, often due to similar colors and specular reflections occurring in the natural environment.

In contrast to the existing approaches, specifically tailored for indoor environments and stationary cameras, in this work, we are interested in developing a laser spot tracking method for an outdoor environment recorded with a moving camera. To the best of our knowledge, no work with this interest has been presented. However, strong interest for laser spot tracking in indoor environments and its potential application for different outdoor laser-camera interactive systems (e.g., laser-guided robots, unmanned aerial vehicles, shooting training evaluation and targeting) motivated the development of the outdoor laser spot tracking method. We believe that the proposed laser spot tracking method could encourage the research community to investigate its potentials for different outdoor laser-camera guidance systems and help to improve existing indoor environment applications, as well. In order to develop a functional system, a number of challenges need to be met. The major challenges of tracking a laser spot in images in an outdoor environment obtained by a non-stationary camera are attributed to uncontrolled conditions and camera movements. In uncontrolled conditions, bright lighting (e.g., sunlight) can impair the visibility of the laser spot, while different materials reflecting natural light and a laser beam from its surface can produce phantom spots, thus presenting a confusing factor for targeted laser spot tracking. A moving camera introduces additional laser spot jitter, besides that existing due to natural hand tremor present during the laser pointing gesture. Additionally, when the camera is moving, the background of the recorded image is moving, as well, and the motion of the laser spot should be distinguished from the background motion without any assumption on the velocity and acceleration of the motion of the camera. Furthermore, usual difficulties encountered during tracking in an indoor environment with a static camera, such as the disappearance and reappearance of the target and cluttered backgrounds, are still present. In regard to enumerated specificities, a robust algorithm is required that is computationally efficient and has the potential to run in real time. In the following, we will present and extensively evaluate a method for tracking a laser spot from a non-stationary camera in an outdoor environment based on modified circular Hough transform [16] and motion pattern analysis, which achieves encouraging results.

Two major contributions of this paper are: (i) the proposition of a novel method for laser spot tracking in an outdoor environment with real-time performance, which is also highly accurate in an indoor environment when detecting a laser spot; (ii) an extensive database with ground truth data is collected and made publicly available. The rest of the paper is organized as follows. In Section 2, the proposed method is given in detail. Section 3 describes evaluation data and discusses the evaluation strategy. Evaluation results are presented in Section 4. The paper is concluded with Section 5.

## 2. Laser Spot Detection and Motion Pattern Analysis Method

**Figure 1.**
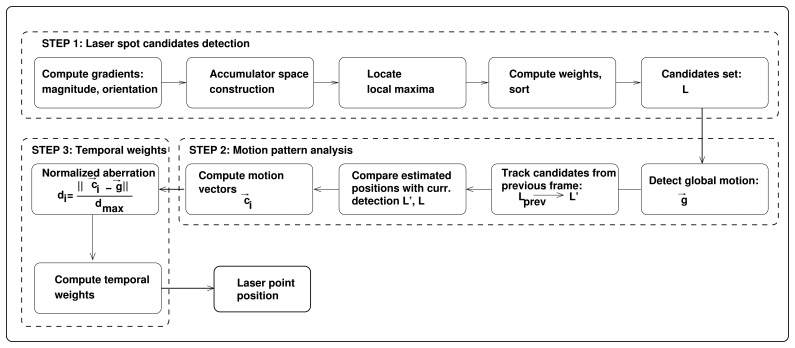
Laser spot tracking based on modified circular Hough transform (mCHT) and motion pattern analysis.

Before proceeding to the description of the method, we would like to emphasize the difference between detection and tracking of the laser spot, as these are used ambiguously in the literature of laser spot tracking. Detection is a process of identifying the presence of the laser spot in a single image and can be regarded as a spatial operation, while tracking is a process of continuous identification of the laser spot in a sequence of images. Usually, laser spot tracking is achieved by concatenating sequential detections, without exploiting any temporal relation between consecutive frames. This is known as a tracking-by-detection approach. In this framework, false detections, a crucial problem of laser spot tracking according to [8], cannot be properly addressed without delay, which is unsuitable for real-time applications. Here, concatenation of detections is performed over a larger temporal window, *i.e.*, detections from future frames are used to locate true detection in the current frame with temporal delay [17]. When tracking is performed by exploiting the motion pattern of a tracked object in a frame-by-frame manner, quality information is added to help the decision process. Our method exploits both spatial and temporal characteristics of images, which offers a scheme to reduce and eliminate false positive detections, thus enabling correct tracking of a laser spot. In a number of approaches dealing with the small target tracking problem, similar spatio-temporal analysis has been successfully followed [18–20]. Our laser tracking method jointly combines detection and tracking into a framework comprised of the following three steps shown in Figure 1:

*Step 1*. Laser spot candidates detection based on modified circular Hough transform: this step aims to detect a laser spot by revealing regions with a high density of intersections of gradients directed from a darker background to brighter objects. Due to the natural environment, small round-shaped bright objects are highly likely to appear, which will result in a number of laser spot candidates.
*Step 2*. Motion pattern analysis: Lucas–Kanade tracking of two independent point sets is required in order to distinguish between false detections originating from the background and detection belonging to the true laser spot. Here, the assumption is made on the motion pattern of the laser spot and the motion pattern of the background.
*Step 3*. Combining the outputs of detector and motion pattern analysis: To estimate the final location of the laser spot, the fusion of weighted detections and motion analysis votes is performed.

The unique characteristic of our approach is the use of a detector based on modified circular Hough transform (mCHT) and motion pattern analysis. Our detector uses both shape and brightness characteristics of a laser spot in terms of processing image gradients and assigning weights to candidates. In state-of-the-art detection methods, different schemes are exploiting only one feature of a laser spot, such as appearance (in template matching), grey level values (in brightness thresholding) or color (in color segmentation). Furthermore, in state-of-the-art methods, the motion trajectory of the laser spot is obtained by memorizing sequential detections. Our motion pattern analysis uses the assumptions of the Lucas–Kanade tracker to jointly analyze the motion of the laser spot and the motion of the background, thus allowing motion compensation, which, in turn, eliminates false positives.

While the proposed method can be used on standard color and monochrome images for indoor and outdoor scenarios, in outdoor environments, abundant speckle noise occurs, which easily confuses the algorithm in detecting and tracking the targeted laser spot. To improve robustness in outdoor conditions, we mounted a bandpass optical filter on camera. In our experiments, we use a 532-nm green laser pointer with a <5 mW output power coupled with the camera equipped with a band-pass optical filter with a 532-nm central wavelength.

### 2.1. Laser Spot Candidates Detection Based on Modified Circular Hough Transform

**Figure 2.**
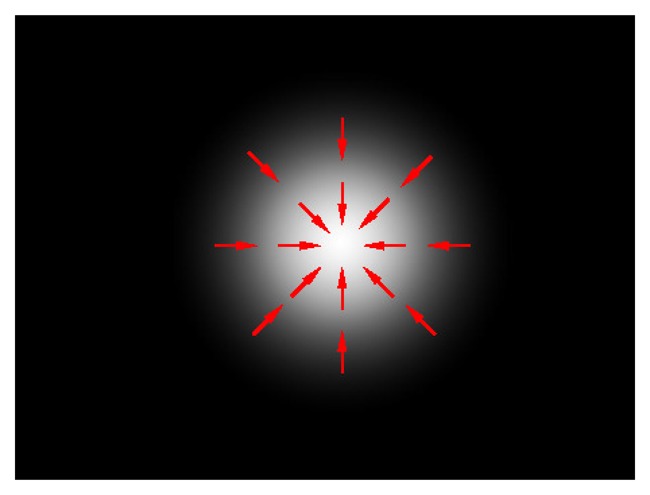
Ideal laser spot; gradient directions are shown in red.

**Figure 3.**
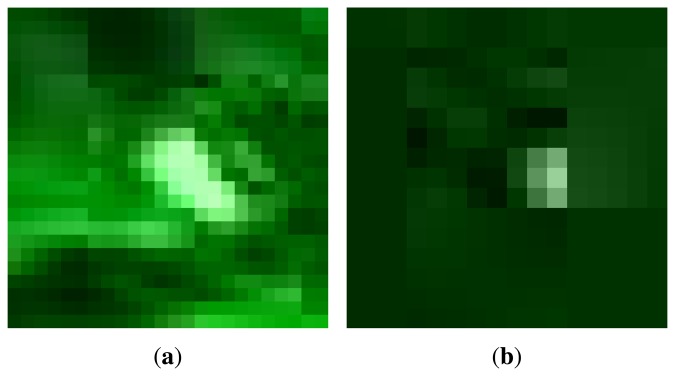
Magnified examples of real laser spots. (**a**) Moving laser spot on an irregular surface; (**b**) laser spot at a long distance.

Detection of the laser spot, as the first step of our method, is based on modification of the circular Hough transform (CHT) [16] algorithm. CHT enables the extraction of circles characterized by a center point (*x_c_, y_c_*) and a radius *r*. The CHT will detect spots with higher brightness in places where centers of round-shaped objects should be found. An ideal laser spot is represented by a white spot in the input image, *i.e.*, a rapid change from a dark background to the local maxima, as shown in Figure 2. However, the assumption that the laser spot is represented by an ideal circle holds only when the spot is static and relatively close to the camera. In real-life scenarios, a laser spot can be moving fast, resulting in the deformed image of a laser spot. Further, when a laser spot is at a longer distance from the camera, it only occupies a few pixels of an image and has an irregular shape. Examples of real laser spots are shown in Figure 3. While CHT can be used for the detection of round shapes whose outlines are not deformed, such as in [21], to efficiently detect a laser spot, the original CHT should be modified in such a way as to relax the assumption of CHT, which states that the gradient orientation will be uniformly distributed and directed at one point. The modification made for the purpose of irregular spot detection (or any small bright object) is described in the following.

The reasoning of our technique is to rely on detecting regions of higher density gradient intersections directed from a darker background to the brighter mid-area of small objects. When such a region is detected, the contribution of all gradients in the neighboring area is superimposed on the weight of the detected candidate, since a laser spot in a real-life scenario is generally not represented by a single point of intersection of gradients. The outline of the proposed technique for identifying laser spot locations is given in Algorithm 1.

First, the gradients of an image *I* are computed:

(1) $$\nabla I(x,y)=\left(\frac{\partial I(x,y)}{\partial x},\frac{\partial I(x,y)}{\partial y}\right)$$

(2) $$g_{x}(x,y)=\frac{\partial I(x,y)}{\partial x},\;g_{y}(x,y)=\frac{\partial I(x,y)}{\partial y},$$

where *g_x_* represents the gradient in a horizontal and *g_y_* in a vertical direction. In the region of the laser spot, Equation (2) yields a set of gradient vectors spanning from the outskirts to the mid-area of the laser spot.

The computed gradients are transformed to polar coordinates, where $mag(x,y)=\sqrt{g_{x}^{2}+g_{y}^{2}}$ is the magnitude of the gradient on pixel coordinates (*x, y*) and *α*(*x, y*) is its orientation. Gradients with *mag*(*x, y*) < *Th*, where *Th* is a predefined threshold value, are set to zero to remove noise introduced by weak transitions in the input image. The accumulator space *acc* of the same dimensions as the input image is then created. For each gradient with a magnitude above a given threshold *Th*, a set of candidates for the associated laser spot center is computed:

(3) $$\begin{array}{lll} x_{c} & = & x+r\cos[\alpha (x,y)]\\ y_{c} & = & y+r\sin[\alpha (x,y)]\\ r & = & 1,\ldots ,R \end{array}$$

where (*x_c_, y_c_*) is a candidate location for the spot center and radius *R* is defined as the maximum expected radius of the laser spot. The contribution of each gradient with a magnitude above threshold *Th* is added to all possible candidate centers (*x_c_, y_c_*) up to distance *R*.

Finally, candidates for the laser spot centers are selected based on two criteria: local maximum in the accumulator space and associated local maximum in the input image, which is less than *R* distant from the location of the local maximum in the accumulator space. If both criteria are satisfied, the laser spot candidate is added to the candidate list with weight:

(4) $$w=\sum_{i=x-R}^{x+R}\sum_{j=y-R}^{y+R}acc(i,j)$$

computed as the sum of all accumulator space values in distance *R* from the location of the peak. Candidates are sorted according to their weights, resulting in a set of the possible laser spot locations:

(5) $$L=\{l_{1},\ldots ,l_{n}\},$$

where *l*_1_ is the is most likely location of the laser spot and *n* is the cardinality of the *L*.



**Algorithm 1** Detect laser spot candidates.
**Require:** Image *I***Require:** Parameter *R* *g_x_, g_y_* ← *I* *mag, α* ← *g_x_, g_y_* *acc* ← *createEmptySpace*(*size*(*I*)) **for all** (*x, y*) **do**  **if**
*mag*(*x, y*) > *Th*
**then**   **for**
*r* = 1 : *R*
**do**    *x_c_* = *x* + *r* cos [*α*(*x, y*)]    *y_c_* = *y* + *r* sin [*α*(*x, y*)]    *acc* [*x_c_, y_c_*] + +   **end for**  **end if** **end for** *L* ← *createEmptySet* **for all** (*x, y*) **do**  **if**
*is Peak*(*x, y*) in *acc*
**and**
*is Peak*(*x, y*) in *I*
**then**    
$w=\overset{x+R}{\underset{i=x-R}{\text{∑}}}\overset{y+R}{\underset{j=y-R}{\text{∑}}}\text{acc}(i,j)$   *L* ← *addCandidate*[(*x,y*),*w*]  **end if** **end for** *sortByWeigth*(*L*)


**Figure 4.**
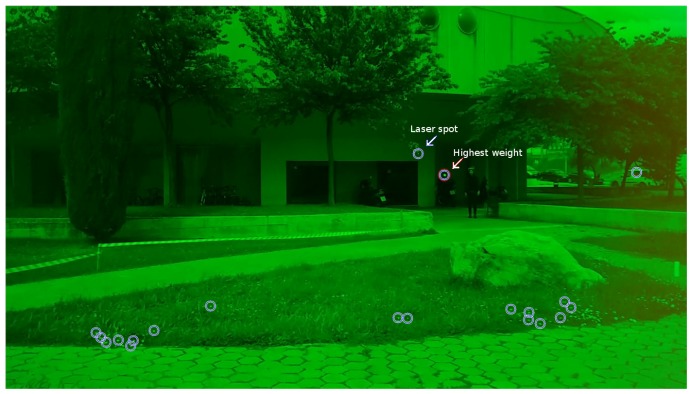
Detected laser spot candidates: the location of the true laser spot and the location of the candidate with the highest weight are marked with arrows.

According to experiments, the first candidate in *L* coincides with the true laser spot with a high probability when the distance between the camera and the spot is relatively small (5 to 10 m). At longer distances, the quality of the detection deteriorates, due to the detection of objects that look similar to the laser spot. This is especially the case in bright sunlight with spot reflections occurring on small reflecting surfaces when these objects are added to the list of candidates. If these reflections are bright and close to the camera, the computed weight for such an object can be higher than the weight of the true laser spot, which is far from the camera. A typical example of detection results is shown in Figure 4. Locations of the true laser spot and the location of the candidate with the highest weight are marked with arrows. The detected laser spot candidate with the highest weight is a sunlight reflection on the rear-view mirror of a parked motorcycle. The actual position of the laser spot is also included in the candidate list as a candidate with lower weight. Other detections, *i.e.*, false detections that are included in the list, are bright and small objects near the camera.

### 2.2. Motion Pattern Analysis

Motion pattern analysis of detected candidates and global image features is performed with the intention of boosting up the weight of the true laser spot. This part of our method is based on the assumption that the motion pattern of most of the detected objects is a consequence of the movement of the camera, which differs from the motion pattern of the laser spot. Further, very small objects, e.g., reflections, which look like a laser spot, tend to be unstable, due to changes in illumination conditions and camera orientation. The motion pattern analysis algorithm favors candidate detections that can be consistently tracked in consecutive frames and whose motion pattern does not coincide with the movement of the camera.

Motion pattern analysis, outlined in Algorithm 2, is based on tracking two sets of points. The first set of points, tracked between consecutive frames, estimates the global motion vector representing the motion of the camera. The second set of points is comprised of candidate detections from the previous frame that will be tracked to estimate their new location. Ultimately, this will enable the selection of consistent detections by the comparison of candidate detections tracked from the previous frame with the candidate detections obtained in the current frame.

The first set of points is acquired by identifying points with distinctive features, based on the Shi-Tomasi algorithm [22]. An extracted set of sparse points from the previous frame is tracked by pyramidal Lucas–Kanade [23,24] to determine the location of the tracked points in the current frame. For each successfully tracked point, the distance and orientation of the motion vector is calculated. Ordered set $S=\left\{\vec{s_{1}},\ldots ,\vec{s_{m}}\right\}$ is formed by sorting vectors by orientation, where *m* is the number of successfully tracked points. After sorting, the mid-third of tracked points is selected, based on the assumption that the objects with their own moving pattern would be on the tails of the distribution, while the points in the middle of the distribution have a moving pattern that is a consequence of the camera movement, yielding set *S*′ = *S*(*m*′ : 2*m′*), where *m′* = *m*/3.

The remaining motion vectors in set *S′* are sorted again by the distance traveled between frames, and the global movement vector *g⃗* is selected as the median of the sorted set *S′*.

A second set of tracked points is a set of *K* candidates *L_prev_* = {*p*_1_,…,*p_K_*} from the previous iteration, *i.e.*, laser spot candidates detected in the previous frame *I_prev_*. Pyramidal Lucas–Kanade is used to estimate new positions $\tilde{L}=\{\tilde{p_{1}},\ldots ,\tilde{p_{K}}\}$ of tracked candidates in the current frame. A set of laser spot candidates *L* detected in the current frame *I* is compared to the successfully-tracked points in *L̃*. For each candidate *l* ∈ *L* with a corresponding candidate in *L̃* displacement vector *c⃗_i_* is computed as the difference between previous and current positions. For candidates with no corresponding detection in the previous frame, the displacement vector is set to global movement vector *c⃗_i_* = *g⃗*.



**Algorithm 2** Motion pattern analysis and temporal weights computation.
**Require:** Candidate sets *L* = {*l*_1_, …,*l_n_*}, *L_prev_* = {*p*_1_, …,*p_K_*}**Require:** Frames *I, I_prev_***Require:** Parameters *C*_1_, *C*_2_ *g⃗* ← *detect Global Motion*(*I_prev_, I*) *L̃* = {*p̃*_1_, …, *p̃_k_*} ← *estimateCurrentPositions*(*L_prev_, I_prev_, I*) **for all**
*l* ∈ *L*
**do**  **if**
*l* = *p̃*(*k*) ∈ *L̃*
**then**   *c⃗_i_* = *estimateMotionVector*(*p*(*k*), *l*)  **else**   *c⃗_i_* = *g⃗*  **end if** **end for**  
$d_{\max}=\underset{i=1,\ldots ,n}{\arg\max}\left\|{\vec{c}}_{i}-\vec{g}\right\|$ **for all**
*l* ∈ *L*
**do**   
$d_{i}=\frac{\left\|{\vec{c}}_{i}-\vec{g}\right\|}{d_{\max}}$  **if**
*d_i_* > 0 **then**   *t_i_* = *C*_1_*d_i_w_i_*  **else**   *t_i_* = *C*_2_*w_i_*  **end if** **end for**


### 2.3. Combining Outputs of Detector and Motion Pattern Analysis

For each candidate in set *L*, normalized aberration is computed:

(6) $$d_{i}=\frac{\left\|{\vec{c}}_{i}-\vec{g}\right\|}{d_{\text{max}}}$$

where:

(7) $$d_{\max}=\underset{i=1,\ldots ,n}{\arg\max}\left\|{\vec{c}}_{i}-\vec{g}\right\|$$

is the maximum aberration between global move *g⃗* and displacement vectors *c⃗_i_*, and *n* is the number of candidates in set *L*. The temporal weight *t_i_* of the *i*-th candidate is defined as:

(8) $$t_{i}=\{\begin{array}{cc} C_{1}d_{i}w_{i} & \text{for}d_{i}>0,\\ C_{2}w_{i} & \text{for}d_{i}=0 \end{array}$$

where *w_i_* is a candidate detection weight, while *C*_1_ and *C*_2_ are constant factors. By setting *C*_1_ ∈ [2,4], candidates with their own moving pattern that differs from the global motion are favored, while a small value of *C*_2_ ∈ [0.1,0.3] will lower the weight of unstable candidates that appear randomly on some frames. According to Equation (8), coefficient *C*_2_ is also applied to the candidates that can be tracked from the previous frame with displacement vector *c⃗_i_* = *g⃗*. This assures that these candidates have temporal weight *t_i_* > 0. On our evaluation dataset, which will be presented in detail in Section 3, the average number of new candidates (with no motion history) per frame is 2.1, while the average of 4.4 candidates are successfully tracked between consecutive frames. For tracked candidates, an average of 0.7 candidates per frame have their displacement vector *c⃗_i_* = *g⃗, i.e.*, 0.7 tracked candidates per frame satisfy condition *d_i_* = 0. As the last step of the algorithm, candidates are sorted based on their temporal weights, and the first candidate is chosen as the detected location of the laser spot.

In some applications, a laser pointer is used to mark a fixed object. As a result, the true laser spot will be static on an object and will exhibit the motion pattern associated with camera motion, *i.e.*, it will approximately match the global motion vector *g⃗*. In these particular applications, coefficients *C*_1_ and *C*_2_ should be selected to compute temporal weight *t_i_*, which will favor candidates with a motion pattern that matches the global motion *g⃗* (higher weight for lower *d_i_*). Accordingly, in applications when a laser pointer is used to mark a fixed object, the displacement vector *c⃗_i_* of newly detected candidates that cannot be tracked from previous detections can be set to zero, which should result in relatively high *d_i_* and lower temporal weight *t_i_*.

### 2.4. Algorithm Complexity

The proposed algorithm is expected to run in real time on devices with limited resources; thus, the complexity of the algorithm is very important. Laser spot detection starts with gradient estimation based on edge detection with computational complexity *O* (*k*^2^*N*), where *N* is the number of image pixels and *k* is the kernel width, followed by transformation of *N* computed gradients to the polar coordinates. The complexity of the accumulator space construction is *O* (*Rm*), where *m* < *N* is the number of gradients above threshold *Th* and *R* is the maximum expected laser spot radius. The last step in identifying laser spot candidates includes inspection of all *N* image pixels to detect peaks with complexity *O* (*N*). A sorting algorithm is performed on *n* detected candidates with *n* ≪ *N*. On our evaluation dataset, presented in Section 3, the proposed algorithm yields the average number of 6.5 candidate detections per frame. The average number of candidate detections for different distances is given in Table 1. According to these results, in all experiments, the number of candidate detections is limited to 20 and does not introduce a considerable complexity overhead.

The complexity of the Lucas–Kanade optical flow computation, which is the main part of the motion pattern analysis algorithm, is also linear, as shown in [24]. Thus, the overall computational complexity of the proposed algorithm is linear and scales well with the change in the resolution of the processed input stream.

## 3. Dataset Description and Evaluation Methodology

The evaluation dataset is compiled such that representative scenarios for different detection conditions are included. A set of video sequences was recorded in different backgrounds, environmental light conditions and with different distances of laser spot from the camera. All dataset sequences were filmed with a camera on which a narrow-band optical filter was mounted with the central wavelength of the filter matching the laser pointer wavelength. Filter characteristics are given in Table 2. For each sequence, the ground truth (GT) location of the laser spot was manually marked on one frame per second, *i.e.*, on a 30-s sequence, the GT laser spot position was marked on 29 frames equally distributed in time (the first frame marked at *t* = 00:01 and the last frame marked at *t* = 00:29). As the laser spot typically has no regular shape, as shown in Figure 3, it is usually difficult to manually extract the exact location of the center of the laser spot. Thus, the detection is considered correct if the distance between the detected and GT coordinates of the laser spot is less than *R*, where *R* is an algorithm parameter representing the maximum expected radius of the laser spot (3). To ensure objective evaluation, sequences were filmed during several days with different atmospheric conditions (from cloudy to sunny), in a diverse and uncontrolled environment with high human activity. All sequences used in this evaluation accompanied by their GT files are publicly available [25].

**Figure 5.**
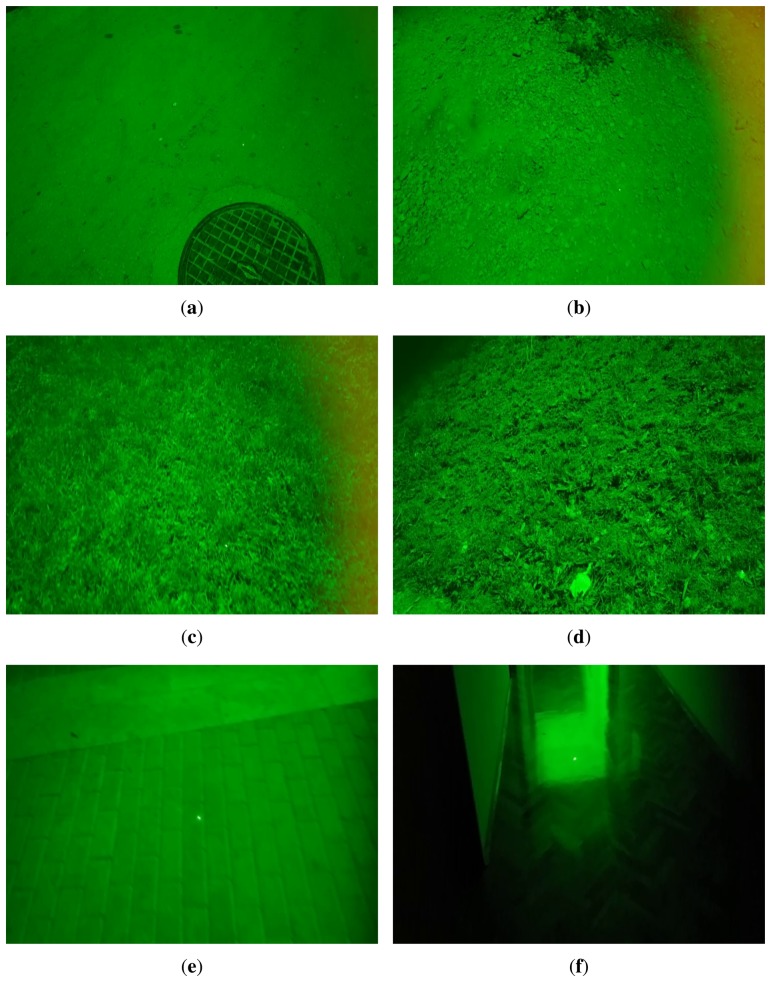
Examples of different backgrounds. (**a**) street; (**b**) dirt; (**c**) fairway; (**d**) grass; (**e**) paver; (**f**) indoor.

The evaluation dataset was divided into two subsets. The first subset contains sequences with different laser spot to camera distances, *i.e.*, 5, 10 and 40 m. On each of the three distances, the tag was installed at the predefined distance (5, 10 or 40 m) and the laser pointer was directed in the region close to that distance. Distance defines the maximal laser spot to the camera distance where the actual distance can be up to the maximal distance. On all sequences, most of the time, the actual distance is close to the maximal distance. Occasionally, the laser spot moves beyond the maximal distance to the background in open space, as the laser pointer moves away from objects near the maximal distance. The second subset contains sequences with different backgrounds, denoted as street, dirt, fairway, grass and paver. Furthermore, indoor sequences are included and denoted as indoor. The street sequence contains images of an asphalted pathway with people walking on it. The dirt sequence contains images of a pathway made of small stones, sand, occasional small plants and other discarded objects, with people occasionally passing by. The fairway sequence contains images of an open space area with cultivated and low-cut grass. The grass sequence contains images of an open space area with uncultivated wild grass and small wild plants. The paver sequence contains images of an area covered with paving stones. The indoor sequence contains images of an indoor faculty area, especially halls, stairs, walls with doors, shiny floors with lots of laser reflections and with people occasionally passing by. Examples of each category are given in Figure 5. In all video sequences in the second subset, the laser spot to camera distance is limited to 5 m to assess the performance of the proposed technique with respect to the background type with no other influences.

During the sequence acquisition, the camera has been handheld by three different persons walking at different walking speeds. the same person was holding the camera and the laser pointer when acquiring the dataset at different distances, while for the acquisition of sequences with different backgrounds, one person was holding the camera and the other was holding the pointer. In all sequences, a certain number of frames is present where the laser spot is occasionally occluded due to scene obstacles (e.g., grass, ground object, walking persons) or temporarily not present due to leaving and entering the field of view of the camera.

Evaluation was divided into two phases. First, the efficiency of the laser spot detection based on mCHT, described in Section 2.1, is evaluated. Secondly, the performance of the overall method, i.e., the mCHT-based detector with motion pattern analysis, as described in Section 2.3, was evaluated. This evaluation approach gives particular insight into the contribution of the motion pattern analysis step, described in Section 2.2. Please note that in all tables and figures in Section 4, which follows, detection denotes a detector based on mCHT and detection with motion analysis denotes the overall method, *i.e.*, a detector based on mCHT with motion pattern analysis.

## 4. Evaluation Results

### 4.1. Results on Varying Distances

To evaluate the efficiency of the proposed technique with respect to the proximity of the laser spot to the camera, sequences were recorded with the maximal distance of the laser spot to the camera set to 5, 10 and 40 m. In total, there are 12 sequences, *i.e.*, four sequences per each of the three distances. Each sequence is 30 s long.

**Figure 6.**
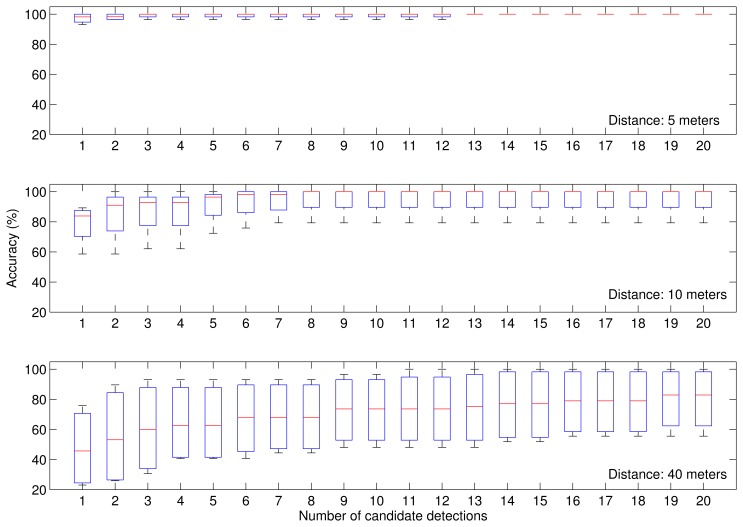
Percentage of correct detections of order *n* of the mCHT-based detector.

Results for mCHT-based detection without motion pattern analysis are given in Table 3. Each column represents the percentage of correct detections of order *n* in all input images, where order *n* = *k* means that the actual position of the laser spot is included in the candidate list in the first *k* candidates. As expected, the accuracy of the detection degrades with the distance from the camera. The laser spot is correctly detected as the first candidate in almost all images when the distance between the camera and the laser spot is not more than 5 m. When the laser spot is at a distance of up to 10 m from the camera, the accuracy of the first detection decreases to 79%. For even longer distances of up to 40 m, the true laser spot is correctly detected as the first candidate in less than 50% of all detections. However, there is a high probability that at longer distances, the laser spot will be detected as a candidate with a lower weight. At distances up to 40 m, the laser spot is included within the first 20 candidates with 80% probability. A boxplot representation of results in Figure 6 gives the overall distribution of the detection accuracy of order *n* for the first 20 candidates, where the red line is the median, the ends of the box are quartiles and the whiskers are the extreme values.

**Figure 7.**
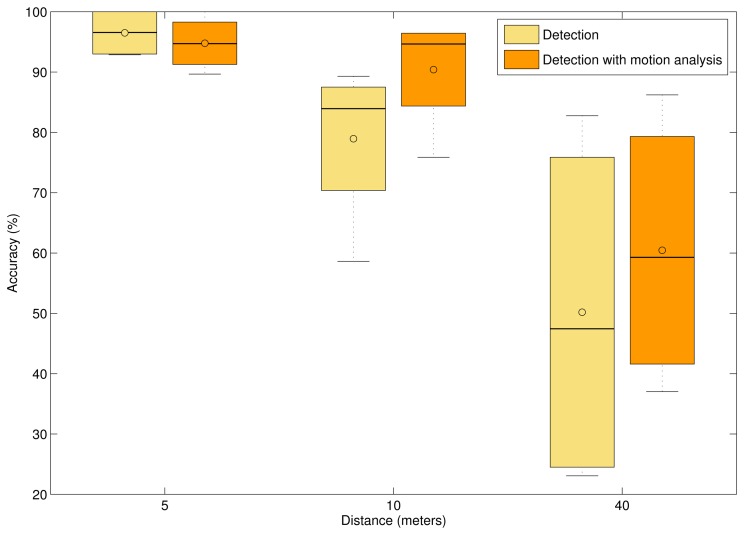
Laser spot detection accuracy for the mCHT-based detector (yellow boxplots) and for the mCHT-based detector and motion pattern analysis (orange boxplots) for linebreak different distances.

In Figure 7, the accuracy of laser spot detection for the mCHT-based detector (yellow boxplots) and mCHT based detector with motion pattern analysis (orange boxplots) for different distances is given. The line within the box represents the median value, and ○ represents the mean value. Detection results for sequences with a laser spot at a distance of up to 5 m from the camera are given in Table 4. It is interesting to point out that at close range distances, when the laser spot is less than 5 m from the camera, mCHT-based detection yields 96% average accuracy and motion analysis does not improve the results. In fact, temporal analysis slightly degrades overall performance. At close distance ranges, the laser spot is usually detected as the first candidate by the mCHT-based detector with high detection weight *w*. The motion of objects at a close distance can, in some cases, degrade the tracking ability of the motion pattern analysis, decrease the temporal weight of the true laser spot and increase the temporal weight of false positive detections. More discussion on this will be given in Section 4.4.

For distances up to 10 m, given in Table 5, motion pattern analysis improves the overall mean accuracy by almost 15%. Improvement introduced by motion pattern analysis increases over 20% for long range detection, as shown in Table 6. From the results, it can be observed that motion pattern analysis gives robustness to the method when the laser spot is at longer distances for which the number of false detection candidates provided by the mCHT-based detector increases (Table 1).

### 4.2. Results on Different Surfaces

**Figure 8.**
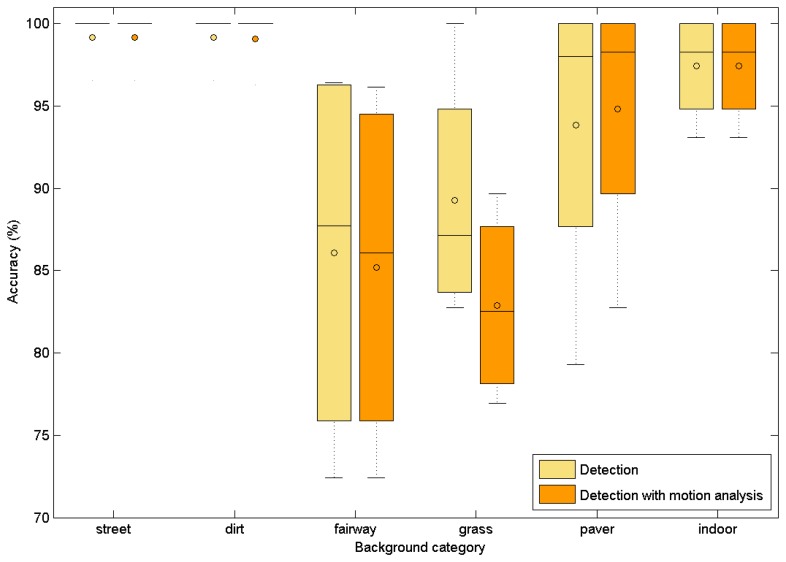
Laser spot detection accuracy for the mCHT-based detector (yellow boxplots) and for the mCHT-based detector and motion pattern analysis (orange boxplots) for different backgrounds.

The performance of the proposed algorithm on different backgrounds is evaluated in the following. The algorithm was tested on five different background surfaces in different daylight conditions. A total of 20 sequences was recorded, four for each background type. In all sequences, the maximal laser spot to the camera distance was kept under 5 m. Additionally, the method was tested on four indoor sequences. The results for the correct first detection are given in Figure 8. The mean value of the laser spots correctly recognized as the candidate with the highest weight for different surface types is given in Table 7. The algorithm achieves acceptable results on all surface types. It can be observed that surface irregularity affects the algorithm performance. Lowest performance is achieved on grass and fairway where surface irregularity results in more probable occlusions of the laser spot. The proposed approach achieves an average of 97.41% accuracy for indoor tracking. These results are significantly better than the best success rate (true positive) of 69% reported in the study of [8], where template matching and the fuzzy rule method were proposed for laser spot detection. As all sequences were recorded at close range, where the laser spot is less than 5 m from the camera, motion pattern analysis, in some cases, slightly decreased the accuracy of the detection. This is consistent with the results presented in Section 4.1.

### 4.3. Laser Spot Detection and Tracking without Bandpass Filter

Although the proposed algorithm is primarily designed for outdoor environments with uncontrolled environmental light, its performance was tested without a bandpass filter to evaluate the possibility of applying this approach in situations when an optical bandpass filter is not available. Results for sequences in an indoor environment without a bandpass optical filter are given in Table 8. From the top to the bottom, the results are shown for increasing distances, *i.e.*, in sequence01-nofilter, the laser spot is a few meters from the camera, while in sequence04-nofilter, the laser spot is up to 25 m from the camera. The achieved results are consistent with the previous analysis, where motion pattern analysis improves the results at longer distances. The average score achieved with the optical filter for similar indoor sequences is 97.41%, as presented in Table 7. However, in the outdoor environment with bright environmental light, the laser spot is barely visible, even to the human eye, and cannot be detected with the proposed technique.

### 4.4. Discussion

**Figure 9.**
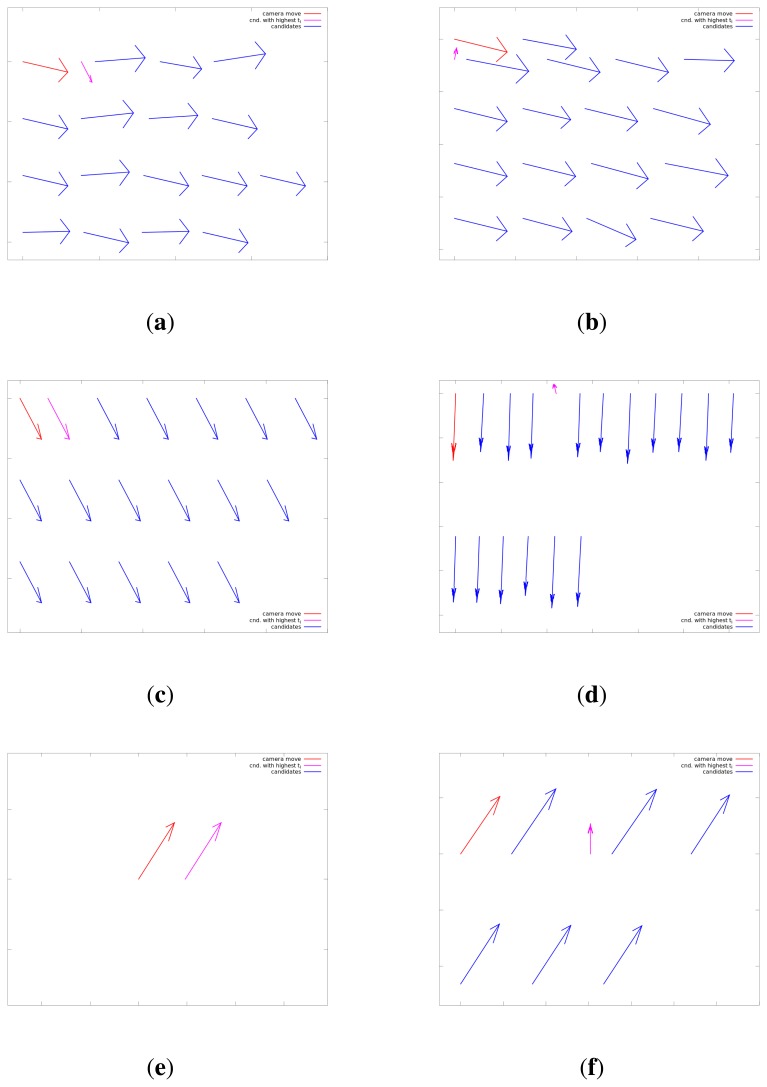
Motion vectors *c⃗_i_* for detections on different distances. (**a**) Motion vectors, max distance 5 m; (**b**) motion vector, max distance 5 m; (**c**) motion vectors, max distance 10 m; (**d**) motion vectors, max distance 10 m; (**e**) motion vectors, max distance 40 m; (**f**) motion vectors, max distance 40 m.

In this section we would like to discuss some specific situations typically encountered in data processing. Motion vectors for six detections of the laser spot at different distances are shown in Figure 9. Global movement vector *g⃗*, corresponding to the camera movement, is shown in red; motion vector *c⃗_i_* of the candidate with highest temporal weight *t_i_*, is shown in magenta, while candidates with lower temporal weights are shown in blue. Vectors are shown in row-major order, sorted by their detection weight *w_i_*. In all subfigures, global movement vector *g⃗* is shown as the first vector in the first row. The second vector in the first row represents the candidate with the highest detection weight *w_i_*, followed by the candidates with lower detection weights sorted in descending order.

On the left side (Figure 9a,c,e), situations are shown where detection weight *w_i_* of the dominant candidate corresponds to the true laser spot. In all three situations, the same candidate has the largest temporal weight *t_i_* (shown in magenta) and is correctly detected as the laser spot.

On the right side (Figure 9b,d,f), situations are shown where the true laser spot is not the dominant candidate according to the detection weight *w_i_* of the mCHT-based detector. Respectively, the laser spot is detected as the candidate with the second largest detection weight at 5 m (first vector in the second row), the fourth detection weight candidate at 10 m (fifth vector in the first row) and the second candidate at 40 m (third candidate in the first row). In all three cases, the true laser spot is correctly recognized as the candidate with the highest temporal weight *t_i_* as a consequence of its distinct motion pattern.

It can be observed in Figure 9a,b, representing typical motion vectors at a close distance range, that the motion pattern of candidate detections not representing the true laser spot are to a certain extent unstable with respect to the global motion vector *g⃗*. These inconsistencies are consequences of the movement of small objects and imaging equipment imperfections when imaging objects at a close range. This phenomena can degrade the tracking ability at close distances in situations when the laser spot is temporarily static on a fixed object and its motion pattern coincides with the global motion vector *g⃗*. These perturbations in the motion vectors of other candidates can boost their temporal weight and confuse the motion pattern analysis algorithm, discarding the true laser spot in favor of a false detection.

The specific case is a scenario where the laser pointer is used to temporarily or permanently mark a fixed object. Examples of these situations are shown in Figure 9c,e. In these situations, the motion vector of the true laser spot candidate does not differ significantly from the motion vectors of the rest of the candidates and vector *g⃗*, and the system relies mainly on the detection weight *w_i_*. If this is a regular scenario for a particular application, the parameters of the algorithm should be fine-tuned to boost the weight of candidates that exhibit the associated motion pattern.

In situations when the approaching angle of the laser spot observer significantly differs from the direction of the laser pointer, which can result in temporal occlusions of the laser spot, redetection of the lost laser spot occurs with a delay of up to a few frames after the laser spot becomes visible again.

## 5. Conclusions

We have proposed a novel method for laser spot tracking that can detect and track laser spots with high accuracy at any distance in the range of up to 40 m. A three-step technique was introduced, which jointly combines detection based on modified circular Hough transform and motion pattern analysis based on Lucas-Kanade tracking. According to the results, the proposed method for automatic laser spot tracking is effective and robust with respect to major factors affecting the performance of visual systems, such as distance from camera, non-uniform backgrounds and changing illumination conditions. The success of the method can be attributed to the diversified conception of the method. In particular, the method covers the spatial and temporal characteristics of laser spot images. For the same reason, robustness to environmental conditions is achieved: motion pattern analysis provides robustness to false detections at longer distances, while the detector ensures redetection of the temporarily lost or occluded laser spot. The proposed method is linear in complexity and can be implemented on limited computational power embedded devices, such as laser-guided robots and drones. The implementation of the proposed algorithm on an android mobile device (Samsung Galaxy Note 3) achieves a processing performance of 8 to 10 frames per second, while on a desktop workstation, a performance of 20 to 24 frames per second is achieved.

The proposed method represents a general technique for laser spot detection and tracking. While the method can be implemented as is, it can easily become a part of more complex algorithms tailored to specific systems. Depending on the purpose of the system, the knowledge and specific rules of the system can be included. For example, if the motion of either (or both) the laser pointer or the laser spot observer (e.g., an unmanned vehicle) are known or could be obtained from different sensing modalities (accelerometers, gyro sensors or similar), this knowledge can be used to adapt the proposed technique to the particular application. This knowledge of specific applications can also be used to reduce the algorithm complexity by reducing the search space.

The proposed technique was extensively tested on the evaluation dataset with the marked ground truth position of the laser spot. The evaluation sequences accompanied by GT data are made publicly available for future work and other researchers.

## Figures and Tables

**Table 1.** Average number of candidates for mCHT-based detection.

**Distance**	**Average Number of Candidates**
5m	2.48
10 m	5.30
40 m	11.87

**Table 2.** Optical filter characteristics.

Central Wavelength (CWL)	532 nm
Central Wavelength Tolerance	2 nm
Full Width-Half max (FWHM)	10 nm
Maximum Transmission	40%
Optical Density (OD)	3

**Table 3.** Percentage of correct detections of order *n* of the mCHT-based detector.

**Number of Candidates**						

**Distance**	**1**	**2**	**3**	**5**	**10**	**20**
5m	97.38	98.25	99.12	99.12	99.12	100
10 m	78.94	85.19	86.95	91.32	94.83	94.83
40 m	47.60	55.45	60.92	64.73	73.01	80.34

**Table 4.** Mean value of correct first detections at distances up to 5 m.

	**Detection**	**Detection with Motion Analysis**
sequence01-dist5m	100%	100%
sequence02-dist5m	93.1%	89.66%
sequence03-dist5m	92.86%	92.86%
sequence04-dist5m	100%	96.55%

average	96.49%	94.77%

**Table 5.** Mean value of correct first detections at distances up to 10 m.

	**Detection**	**Detection with Motion Analysis**
sequence01-dist10m	89.26%	96.43%
sequence02-dist10m	58.63%	75.86%
sequence03-dist10m	85.71%	92.86%
sequence04-dist10m	82.76%	96.43%

average	78.94%	90.4%

**Table 6.** Mean value of correct first detections at distances up to 40 m.

	**Detection**	**Detection with Motion Analysis**
sequence01-dist40m	25.93%	37.04%
sequence02-dist40m	23.08%	46.15%
sequence03-dist40m	82.76%	86.21%
sequence04-dist40m	68.97%	72.41%

average	50.19%	60.45%

**Table 7.** Mean value of the correct first detection for different surfaces.

**Background**	**Detection**	**Detection with Motion Analysis**
street	99.14%	99.14%
dirt	99.14%	99.08%
fairway	86.08%	85.19%
grass	89.26%	82.90%
paver	94.83%	94.83%
indoor	97.41%	97.41%

average	94.31%	93.09%

**Table 8.** The mean value of correct first detections without the optical filter in the linebreak indoor environment.

	**Detection**	**Detection with Motion Analysis**
sequence01-nofilter	100%	96.55%
sequence02-nofilter	89.66%	89.66%
sequence03-nofilter	93.10%	93.10%
sequence04-nofilter	58.62%	62.07%

average	85.35%	85.34%
